# Transcriptome Analysis Reveals the Pivotal Genes and Regulation Pathways Under Cold Stress and Identifies *SbERF027*, an AP2/ERF Gene That Confers Cold Tolerance in Sorghum

**DOI:** 10.3390/plants14060879

**Published:** 2025-03-11

**Authors:** Qijin Lou, Peifeng Wang, Miao Yu, Zhigan Xie, Chen Xu, Shengyu Chen, Hao Yu, Rui Zhang, Guangling Tian, Di Hao, Xianshi Ke, Shuai Yu, Jiajia Zhou, Yao Zhao, Chao Ye, Jiyuan Guo, Haiyan Zhang, Mo Chen, Xingbei Liu

**Affiliations:** 1 Department of Resources and Environment, Moutai Institute, Renhuai 564502, China; 2 Institute of Crop Germplasm Resources, Jilin Academy of Agricultural Sciences, Gongzhuling 136100, China; 3 Rice Research Institute, Guangxi Academy of Agricultural Sciences/Guangxi Key Laboratory of Rice Genetics and Breeding/State Key Laboratory for Conservation and Utillzation of Subtropical Agro-Bioresources, Nanning 530007, China

**Keywords:** AP2/ERF, cold tolerance, RNA-seq, seedlings, sorghum

## Abstract

Low temperature at the seedling stage adversely affects sorghum growth and development and limits its geographical distribution. APETALA2/Ethylene-Responsive transcription factors (AP2/ERFs), one of the largest transcription factor families in plants, play essential roles in growth, development, and responses to abiotic stresses. However, the roles of AP2/ERF genes in cold tolerance in sorghum and the mechanisms underlying their effects remain largely unknown. Here, transcriptome sequencing (RNA-seq) was performed on the leaves of sorghum seedlings before and after cold treatment. Several candidate genes for cold tolerance and regulation pathways involved in “photosynthesis” under cold stress were identified via Gene Ontology (GO) and Kyoto Encyclopedia of Genes and Genomes (KEGG) enrichment. Additionally, the AP2/ERF family gene *SbERF027*, a novel regulator of cold tolerance, was functionally identified through a comprehensive analysis. The expression of *SbERF027* was high in seedlings and panicles, and its expression was induced by low temperature; the cold-induced expression level of *SbERF027* was markedly higher in cold-tolerant accession SZ7 than in cold-sensitive accession Z-5. SbERF027 was detected in the nucleus under both normal and cold stress conditions. In addition, the cold tolerance of *SbERF027*-overexpressing lines was higher than that of wild-type plants; while the cold tolerance of lines with *SbERF027* silenced via virus-induced gene silencing (VIGS) was significantly lower than that of wild-type plants. Further research demonstrated that SNP-911 of the promoter was essential for enhancing cold tolerance by mediating *SbERF027* expression. This study lays a theoretical foundation for dissecting the mechanism of cold tolerance in sorghum and has implications for the breeding and genetic improvement of cold-tolerant sorghum.

## 1. Introduction

Sorghum (*Sorghum bicolor* L. Moench), a cereal crop with a semi-arid tropical origin, is a critically important food and feed source in many parts of the world [1,2]. It is sensitive to low-temperature stress throughout its development. Low temperature is one of the main abiotic factors adversely affecting the germination and seedling vigor of chilling-sensitive crops and limiting their production and geographic distribution [3,4]. The frequency of extreme weather events, including extreme low temperatures, is increasing worldwide, and this has posed a threat to sorghum production [5]. Low temperatures prior to seedling emergence and unpredictable cold snaps shortly after emergence can adversely affect seed viability and seedling development by reducing emergence rates in sorghum, which can result in yield loss and quality reduction [6,7]. Thus, cold stress at the seedling stage greatly limits sorghum production, especially in cooler areas.

Cold tolerance is a complex quantitative trait controlled by multiple genes, which are involved in various regulatory mechanisms and metabolic pathways. Variation in environmental factors impedes evaluations of phenotypes for cold tolerance. The methods for evaluating the cold tolerance of crops have been explored since the 1980s, and these studies have advanced our understanding of the cold tolerance of various crops [8,9,10]. The seedling survival rate (SSR) is a crucial indicator for evaluating cold tolerance before and after cold treatment at the seedling stage in sorghum [8]; three methods (cold tolerance coefficient, comprehensive cold tolerance coefficient, and weighted cold tolerance coefficient) have been used to evaluate the cold tolerance of sorghum germplasms at the germination stage via measurements of five biological traits, the germination rate (GR), germ length (GL), radicle length (RL), fresh germ weight (FGW), and fresh radicle weight (FRW), and this has facilitated the evaluation of the cold tolerance of sorghum at the vegetative stage [11]. The chlorophyll content has also been used to evaluate cold tolerance at the seedling stage in rice and maize [12,13,14]. Three methods, including cold stress in high altitude areas (CS-HAA), cold stress in deep water (CS-DW), and cold stress in a phytotron (CS-PT), have been used to characterize cold tolerance through measurements of the seed setting rate and pollen fertility percent at the booting stage in rice [15]. The use of the above evaluation approach and corresponding indices for cold tolerance facilitate studies of QTLs or genes related to cold tolerance in sorghum and other crops.

Improving the cold tolerance of crop plants, cloning cold tolerance genes, and revealing the genetic basis of natural variation in cold tolerance are major goals of current research [16]. Several quantitative trait loci (QTLs) or candidate genes conferring cold tolerance or associated with cold-related traits have been identified in sorghum via genome-wide association studies (GWASs), linkage-mapping-based QTL detection, genotyping-by-sequencing (GBS), and RNA-seq analysis [2,17,18,19,20,21,22,23,24,25], but the functions of these genes in cold tolerance and the mechanisms underlying their effects have not yet been clarified. Several candidate genes associated with cold tolerance at the reproductive stage were identified on chromosomes 3 and 10 via GWASs of 330 sorghum germplasms, and a few novel temperate-adapted accessions with superior and environmentally stable cold tolerance were identified [2,23]. One marker locus (Locus7-2), shown to be associated with low-temperature germination detected through GWASs, yielded 162,177 single-nucleotide polymorphisms (SNPs) [20]. Previous studies have revealed abundant genetic variability in cold tolerance at the seedling stage of sorghum [19,26,27,28], and several QTLs controlling cold tolerance were found in cold-tolerant accessions; these hotspot regions for cold tolerance could aid molecular-marker-assisted selection in sorghum [17,18,19]. Most QTLs for cold tolerance have been detected by GWASs [2,20,21,22,23]; however, variation in cold tolerance in sorghum could also be detected through bi-parental mapping of various genetic populations, such as recombinant inbred lines and near-isogenic lines [17,19,24,25]. Additionally, reverse genetic methods such as transcriptomics, metabolomics, proteomics, and analysis of orthologous genes have also been important for the discovery of genes involved in cold tolerance in sorghum, such as JAZ family genes involved in the response to cold stress [29]. Two key regulatory genes, *Hsp40s* and *SbHSP10-2*, have been shown to play an important role in the cold stress response [30,31]. The above results provide a molecular basis for studies of cold tolerance in sorghum; however, the functions of key cold tolerance genes and the mechanisms underlying their effects have not yet been elucidated.

APETALA2/Ethylene-Responsive factors (AP2/ERFs), one of the largest families of transcription factors (TFs) in plants, play a crucial role in responses to abiotic stress by directly binding to the specific core *cis*-elements in downstream genes. Several AP2/ERF family genes have been found to regulate cold and drought tolerance and root development via their effects in various regulatory networks in rice [32,33,34,35,36,37,38,39]. In addition, AP2/ERF family genes have been identified in other crops. A total of 214, 218, and 171 genes encoding AP2/ERF proteins have been identified in maize, sugarcane, and foxtail millet, respectively [40,41,42]. In sorghum, 158 ERF genes, including 52 DREBs and 106 ERF subfamily proteins, have been identified, and the structure, phylogenetic distribution, expression, and chromosomal localizations of these genes have been analyzed [43]. The function and genetic and molecular basis of AP2/ERF family members involved in the regulation of cold tolerance have not been studied in sorghum, yet such studies are necessary for clarifying the mechanisms underlying the effects of these genes and the gene regulatory networks mediating cold tolerance in sorghum seedlings.

To identify the pivotal genes and regulation pathways involved in cold tolerance in sorghum seedlings, here, leaves of sorghum seedlings before and after cold treatment were used for RNA-seq. The AP2/ERF family gene *SbERF027*, a positive regulator of cold tolerance, was identified. Expression pattern analysis revealed that *SbERF027* was highly expressed in seedlings and panicles, and its expression was induced by low temperature. The cold-induced expression level of *SbERF027* was markedly higher in SZ7 (cold-tolerant) than in Z-5 (cold-sensitive). SbERF027 was detected in the nucleus under both normal and cold stress conditions. In addition, *SbERF027* positively regulates the cold tolerance in sorghum seedlings, and SNP-911 of the promoter was essential for enhancing cold tolerance by mediating *SbERF027* transcriptional output. Our findings will aid future studies aimed at dissecting molecular mechanisms for the cold tolerance of sorghum as well as the breeding of cold-tolerant sorghum varieties.

## 2. Results 

### 2.1. Cold Tolerance Evaluation

**Figure 1:**
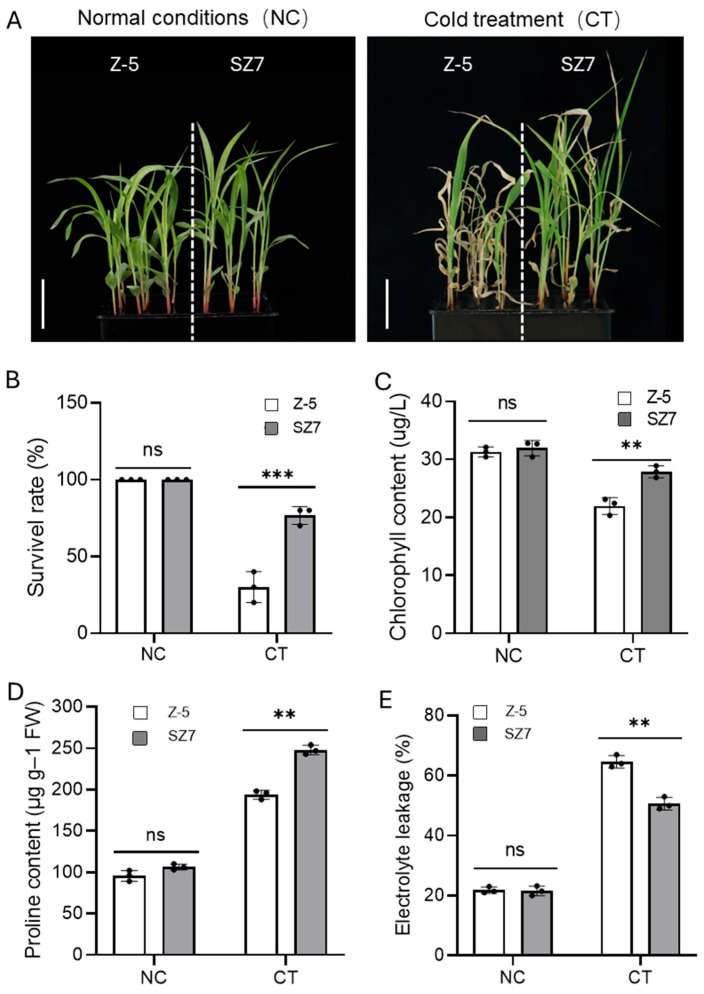
Evaluation of cold tolerance for two sorghum varieties via hydroponic culture. (**A**,**B**) Seedling phenotype (**A**) and survival rate of SZ7 and Z-5 under normal and cold stress conditions; scale bars, 5 cm. (**C**–**E**) The content of chlorophyll (**C**), Pro (**D**), and EL (**E**). Error bars represent standard deviation (SD). Statistical significance was determined by two-tailed Student’s *t*-test: **, *p* < 0.01; ***, *p* < 0.001. ns, no significance.

To identify genes involved in cold tolerance in sorghum seedlings, the cold tolerance of 20 sorghum varieties from different ecological regions was evaluated. Two accessions, Suizhong 7 (SZ7) and Zhong-5 (Z-5), were screened in follow-up experiments. The seedling survival rate of SZ7 was significantly higher than that of Z-5 under cold stress (4 °C for 7 days and recovery for 7 days at 25 °C) (Figure 1A,B and Figure S1). In addition, the chlorophyll content before and after cold treatment was determined, and the chlorophyll content of SZ7 was 1.2 times that of Z-5 after cold stress. The content of proline (Pro) and electrolyte leakage (EL) in the two varieties before and after cold stress was determined. The Pro content of SZ7 is 1.3 times that of Z-5; by contrast, the EL content of SZ7 was 1.28 times that of Z-5. Notably, the three indices mentioned above reached a significant difference level between the two varieties under cold treatment, but there was no significant difference under normal conditions (Figure 1C–E and Figure S1). These results indicated that SZ7 and Z-5 were appropriate materials for identifying genes related to cold tolerance at the seedling stage in sorghum.

### 2.2. Transcriptome Sequencing Data

RNA-seq was performed on 24 samples subjected to low temperature for 0 h (T0), 2 h (T1), 8 h (T2), and 16 h (T3) in the seedling stage, and a total of 124 million raw reads were generated. A total of 116 million clean reads with 177.3 G bases and GC content ranging from 53.8% to 56.3% were obtained after assembly and filtration, and the lengths of the reads ranged from 143 to 144. Additionally, over 97% and 93% of the clean data met the Q20 and Q30 criteria, respectively (Table S1). The average alignment efficiency of the clean reads from the 24 sorghum samples with the reference genome and single exons was 93.4% and 95.5%, respectively (Tables S2 and S3).

**Figure 2:**
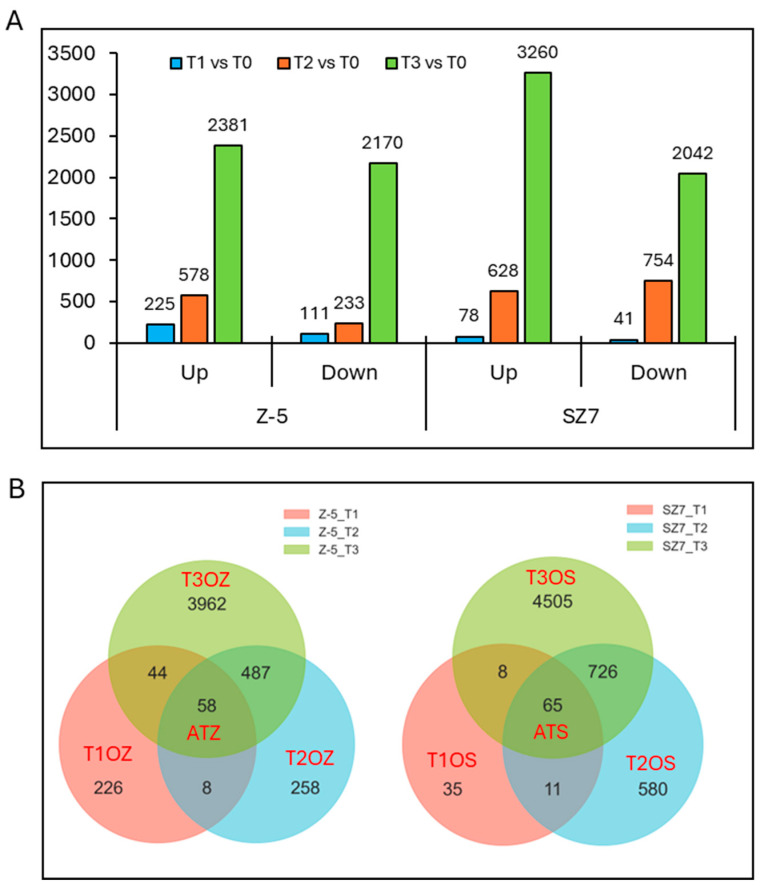
Comparison analysis of DEGs in SZ7 and Z-5. (**A**,**B**) DEG numbers (**A**) and Venn diagram (**B**) of DEGs enriched in two varieties at different time intervals.

Using |log2FoldChange| > 1 and padj < 0.05 as the screening criteria, 5698 and 6803 DEGs were detected in Z-5 and SZ7, respectively (Figure 2A). In the three comparison groups, Z-5_T1 vs. T0, Z-5_T2 vs. T0, and Z-5_T3 vs. T0 (hereafter referred to as Z_T1, Z_T2, and Z_T3, respectively), 336 (225 up-regulated and 111 down-regulated), 811 (758 up-regulated and 233 down-regulated), and 4551 (2381 up-regulated and 2170 down-regulated) DEGs were detected, respectively; a total of 119 (78 up-regulated and 41 down-regulated), 1382 (628 up-regulated and 754 down-regulated), and 5302 (3260 up-regulated and 2042 down-regulated) DEGs were identified in the SZ7_T1 vs. T0, SZ7_T2 vs. T0, and SZ7_T3 vs. T0 comparison groups (hereafter referred to as S_T1, S_T2, and S_T3, respectively) in SZ7, respectively (Figure 2A). These findings indicate that the number of DEGs gradually increased with the duration of cold treatment. We further analyzed DEGs under cold stress in different genotypes, including those specifically expressed at T1 only in Z-5 (T1OZ), at T2 only in Z-5 (T2OZ), at T3 only in Z-5 (T3OZ), at T1 only in SZ7 (T1OS), at T2 only in SZ7 (T2OS), at T3 only in SZ7 (T3OS), and at all time points in Z-5 (ATZ) and SZ7 (ATS). The number of DEGs expressed in T1OZ, T2OZ, T3OZ, T1OS, T2OS, and T3OS was 226, 258, 3962, 35, 580, and 4505, respectively. In addition, a total of 58 and 65 DEGs were identified in ATZ and ATS, respectively (Figure 2B), suggesting that these shared genes may play an important role following the initial exposure of sorghum seedlings to low-temperature stress. The reliability of the RNA-seq data was verified via heat map analysis and quantitative real time-polymerase chain reaction (qRT-PCR) based on FPKM and log2FC data, respectively, which indicated that the sampling process and the RNA-seq data of this experiment have high reliability (Figures S2 and S3).

### 2.3. Functional Annotation of DEGs

To elucidate the functions of the DEGs mentioned above, we performed GO enrichment analysis using a significance threshold of *p* < 0.05 and ListHits > 2 as the significant enrichment criteria. A total of 5464 GO terms were enriched in Z-5. Out of the 5464 GO terms, 348 met the significant enrichment criteria. The number of significantly enriched GO terms in Z_T1, Z_T2, and Z_T3 was 79, 76, and 193, respectively (Figure S4). The most significantly enriched GO terms in Z_T1, Z_T2, and Z_T3 were related to carbohydrate metabolic process (Figure S5, Table S10), proteasome-mediated ubiquitin-dependent protein catabolic process (Figure S5, Table S11), and RNA modification (Figure S5, Table S12), respectively. A total of 5199 GO terms were significantly enriched in SZ7. Out of the 5199 GO terms, 369 terms were significantly enriched, while 73, 130, and 166 GO terms were significantly enriched in S_T1, S_T2, and S_T3, respectively (Figure S4). The three most significantly enriched GO terms were found to play a significant role in the response to water deprivation, response to water, and response to cold (Figure S6, Table S13); thylakoid, plastid envelope, chloroplast thylakoid (Figure S6, Table S14); and protein serine/threonine kinase activity, response to alcohol, response to abscisic acid (Figure S6, Table S15). These results suggested that DEGs enriched in the above GO terms such as “response to cold” might play an important role in the response of sorghum to low-temperature stress.

### 2.4. Analysis of DEGs Enriched in GO Terms Related to “Cold”

**Figure 3:**
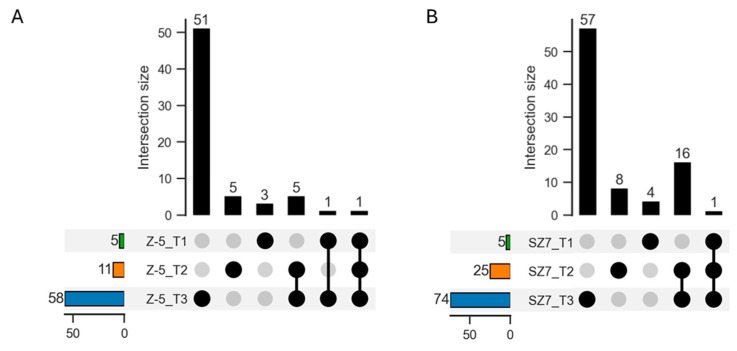
Upset diagram of DEGs enriched in “Response to cold” in Z-5 (**A**) and SZ7 (**B**). The X- and Y-axes mean the enrichment score of DEG numbers enriched in three combinations and gene numbers repeatedly detected in various combinations, respectively. Black solid dots represent the DEG numbers shared by different combinations.

Venn analysis of the significantly enriched GO terms for the different comparison groups revealed only one GO term (GO:0000271) that was shared among the three Z-5 comparison groups, and five shared GO terms were detected among the three SZ7 comparison groups, including GO:0006066, GO:0006109, GO:0009409 (response to cold), GO:0009414, and GO:0009415 (Figure S7). “Response to cold” was one of the shared five GO terms in the SZ7 comparison groups (Figure S7, Table S16). Previous studies have shown that the DEGs enriched in “Response to cold” play an important role in the response to low-temperature stress in rice [5]. Therefore, we analyzed the DEGs enriched in “Response to cold”. A total of 66 DEGs were enriched in the cold-sensitive variety Z-5. In addition, 3, 5, and 51 genes were specifically enriched in T1OZ, T2OZ, and T3OZ, respectively, and only 1 gene encoding a BTB/POZ and TAZ domain-containing protein was enriched in the three Z-5 comparison groups, the function of which remains unknown (Figure 3A, Table S17). A total of 86 DEGs were enriched in “Response to cold” in the cold-tolerant variety SZ7, and 4, 8, and 57 specific genes were enriched in T1OS, T2OS, and T3OS, respectively. Only one gene (*SORBI_3003G425500*), encoding the probable calcium-binding protein CML10, was observed in ATS (Figure 3B, Table S17). A previous study has shown that several CML family members play a role in abiotic stress response processes, such as the response to low temperature in rice [44], suggesting that this gene may play a key role in the response to low temperature or other abiotic stresses.

### 2.5. KEGG Pathway Enrichment Analysis

To identify genes that regulate the response to cold stress, KEGG pathway enrichment analysis was performed using the above DEGs. A total of 2339 and 3445 DEGs were effectively enriched in KEGG pathways in Z-5 and SZ7, respectively (Figure S8). A total of 119 and 203 KEGG pathways were enriched in Z-5 and SZ7, respectively; 3, 6, and 3 KEGG pathways were significantly enriched in Z_T1, Z_T2, and Z_T3, respectively (Figure S9, Table S18); 10 and 5 KEGG pathways were significantly enriched in S_T2 and S_T3, respectively (Figure S10, Table S19). Several of the KEGG pathways mentioned above were related to plant photosynthetic systems, implying that KEGG pathways related to photosynthesis may play a crucial role in the response of plants to low temperatures.

A total of 221 DEGs were significantly enriched in KEGG pathways in the two varieties, including 62 in cold-sensitive Z-5 and 159 in cold-tolerant SZ7, indicating that adaptation to low-temperature stress in cold-tolerant varieties may be improved via activation of the expression of a greater number of DEGs following exposure to cold stress (Table S20). In addition, 4 DEGs were detected in both genotypes, and 138 and 46 DEGs were exclusively detected in one of the two varieties, respectively (Figure S8). Two common genes each were identified in the Z_T3 and S_T2 (*SORBI_3004G074000*, *SORBI_3004G268700*) and Z_T2 and S_T3 (*SORBI_3003G425500*, *SORBI_3005G024300*) comparison groups. One of the four shared genes, *SORBI_3003G425500*, was the same as that identified in the results of the previous analysis of DEGs enriched in “Response to cold”, which encodes a probable calcium-binding protein CML10; the remaining three genes were *SORBI_3005G024300* (a neo-calmodulin), *SORBI_3004G074000* (a lycopene beta cyclase, chloroplastic), and *SORBI_3004G268700* (an abscisic acid 8′-hydroxylase 1). Previous studies have shown that proteins related to calcium binding, chloroplast synthesis, and the abscisic acid metabolic pathway are involved in regulating the response to cold stress [44,45,46]; however, the roles of these genes in regulating the cold tolerance of sorghum have not yet been clarified.

### 2.6. TFs Related to Cold Tolerance

TFs are a group of proteins that bind specifically to exclusive sequences upstream of certain genes to ensure whether they will be turned “on” or “off” at specific times and locations at specific intensities [47,48]. In this study, 746 differentially expressed TFs were detected in both cold-tolerant and cold-susceptible genotypes, which could be divided into more than 30 TF families (Figure S11). Many of these TFs have been reported to be involved in regulating the responses to various types of abiotic stress in sorghum or other crops, such as MYB/MYB-related [49], ERF [46], bHLH [50], WRKY [51], C2H2 [52], and NF [53] TFs.

**Figure 4:**
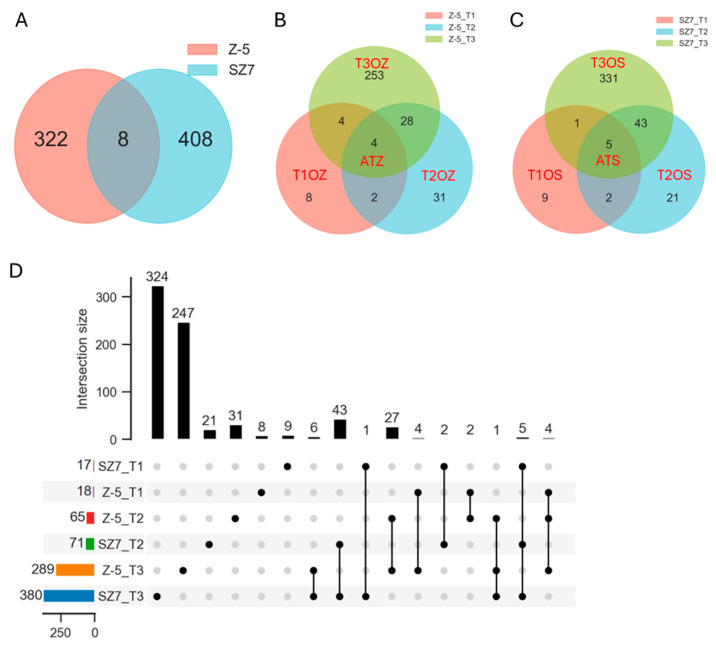
Comparative analysis of TFs enriched in Z-5 and SZ7. (**A**) Venn diagram of TFs between Z-5 and SZ7 in all combinations under cold stress. (**B**,**C**) Venn diagram of TFs enriched in Z-5 (**B**) and SZ7 (**C**) at different time intervals, respectively. (**D**), Upset diagram of TFs between Z-5 and SZ7. Black solid dots represent the number of KEGG terms shared by different combinations.

Comparative analysis revealed 322 Z-5-specific and 408 SZ7-specific TFs, as well as 8 TFs common to both genotypes (Figure 4A). There were 8, 31, and 253 TFs in T1OZ, T2OZ, and T3OZ in the cold-sensitive genotype, and 9, 22, and 331 TFs were detected in T1OS, T2OS, and T3OS in the cold-tolerant genotype, respectively; only 4 and 5 TFs were commonly expressed in ATZ and ATS, respectively (Figure 4B,C). Further comparative analysis between different genotypes revealed a total of 8, 31, 247, 9, 21, and 324 TFs in T1OZ, T2OZ, T3OZ, T1OS, T2OS, and T3OS, respectively, and only 6 TFs were detected simultaneously in both Z_T3 and S_T3; only 1 TF gene was commonly detected in Z_T2, Z_T3, and S_T3 (Figure 4D). This suggested that these genes might be involved in cold tolerance regulation in different phases of the response to low-temperature stress.

**Figure 5:**
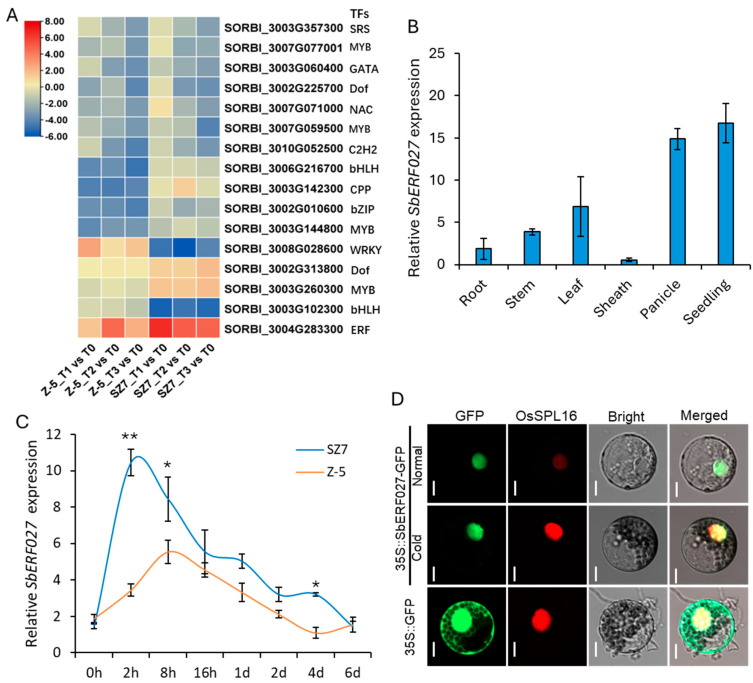
Analysis of heat maps and expression pattern of *SbERF027*. (**A**) Comparison of heat maps for 16 potential candidate TF genes based on the log ratio fold change. The color-coded boxes represent expression levels, with red denoting high expression and blue denoting low expression. (**B**) Tissue expression patterns of *SbERF027* in SZ7 under normal conditions. Error bars represent SD (n = 3). (**C**) Relative expression of *SbERF027* in leaves from SZ7 and Z-5 under cold treatment at different time points. *SbActin1* was used as internal reference. Error bars represent SD (n = 3), Student’s *t*-test, * *p* < 0.05, ** *p* < 0.01. (**D**) Subcellular localization of SbERF027 in wheat protoplasts. Scale bars, 10 um.

The six common TF genes detected in both Z_T3 and S_T3 belonged to the ERF, GeBP, MYB, NAC, and NF-Y families; four common TF genes detected in ATZ belonged to the bHLH, CPP, bZIP, and MYB families; five genes detected in ATS belonged to the WRKY, Dof, MYB, bHLH, and ERF families; and one gene commonly detected in Z_T2, Z_T3, and S_T3 belonged to the C2H2 family (*SORBI_3010G052500*) (Figure 5A, Table S21). Among the 12 TF genes mentioned above, the |log2FC| values of seven genes were greater than 2 in S_T3 and Z_T3; the expression of one gene (*SORBI_3008G028600*) was up-regulated in Z-5 and down-regulated in SZ7. No significant changes in the expression level of two genes (*SORBI_3002G313800*, *SORBI_3003G260300*) were observed in Z-5, but the expression of these genes was significantly up-regulated in SZ7 (Figure 5A, Table S21). Furthermore, the expression of one (*SORBI_3004G283300*) gene was significantly up-regulated in both Z-5 and SZ7, and the expression of this gene was up-regulated to a greater degree in SZ7 than in Z-5, suggesting that *SORBI_3004G283300* might be involved in regulating cold tolerance at different phases under cold stress by altering the expression levels of various genes in sorghum.

### 2.7. SbERF027 Positively Regulates Cold Tolerance in Sorghum Seedlings

**Figure 6:**
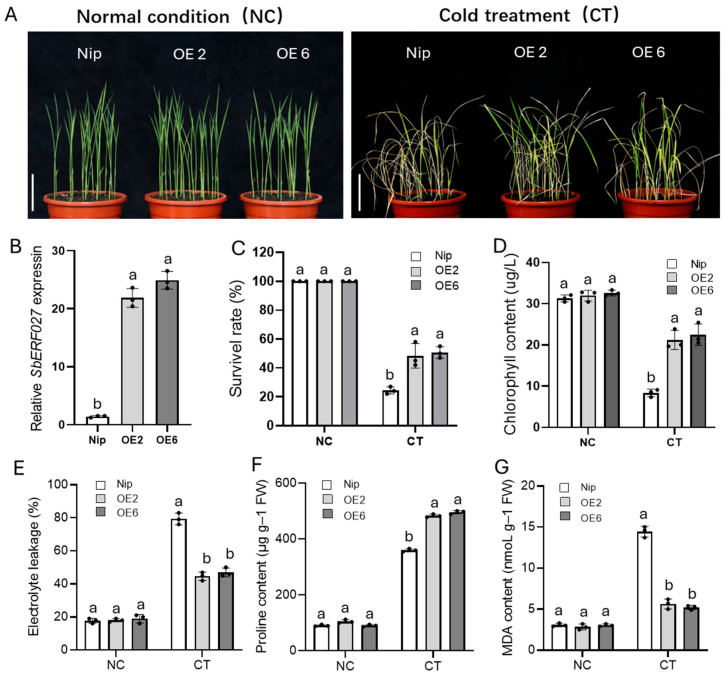
Phenotypic evaluation and determination of physiological indices in *SbERF027*-overexpressing lines in rice. (**A**–**G**) Seedling phenotype (**A**), relative expression of *SbERF027* (**B**), survival rates (**C**), chlorophyll content (**D**), EL levels (**E**), Pro (**F**), and MDA (**G**) for overexpressed lines of *SbERF027* and control plants (Nip) under normal and cold stress conditions. Scale bars, 5 cm. NC, normal condition; CT, cold treatment. Error bars represent SD (n = 3) (Tukey’s test). Significant differences are represented by different lowercase letters above the bars.

**Figure 7:**
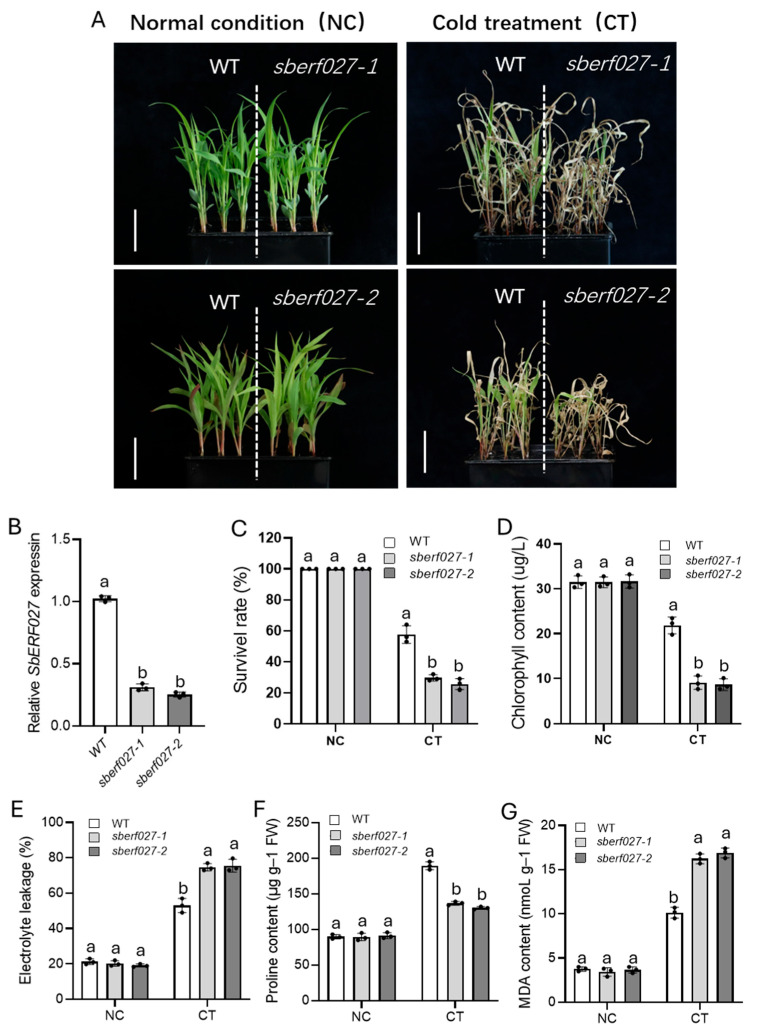
Phenotypic evaluation and determination of physiological indices in *SbERF027*-silenced lines in sorghum. (**A**–**G**) Seedling phenotype (**A**), relative expression of *SbERF027* (**B**), survival rates (**C**), chlorophyll content (**D**), EL levels (**E**), Pro (**F**), and MDA (**G**) for *SbERF027*-silenced lines and wild-type (WT) plants under normal and cold stress conditions. Scale bars, 5 cm. NC, Normal condition; CT, cold treatment. Error bars represent SD (n = 3) (Tukey’s test). Significant differences are represented by different lowercase letters above the bars.

To identify the candidate genes involved in regulating cold tolerance at the seedling stage in sorghum, we performed heat map analysis based on the log2FC data of 16 possible candidate genes from the TF analysis, which were generated from the following comparison groups: Z_T3 and S_T3; ATZ, ATS, and Z_T2; and Z_T3 and S_T3. The expression of one gene, *SORBI_3004G283300*, was higher than that of other candidate genes under cold treatment, and its expression first increased (2 h) and then decreased (8 h, 16 h) as the duration of the cold treatment increased in SZ7; the expression of this gene was significantly higher in SZ7 than in Z-5, and it was first up-regulated at 8 h in Z-5 (Figure 5A). Additionally, several SNPs (SNP-459, SNP-911, and SNP-923) were detected on the promoter between SZ7 and Z-5 via sequence comparison (Figure S12). These results suggest that *SORBI_3004G283300*, which encodes an ethylene-responsive transcription factor ERF027-like protein (SbERF027), is a possible candidate gene for cold tolerance in sorghum seedlings. To clarify the function of *SbERF027*, we overexpressed *SbERF027* in rice and generated two independent *SbERF027*-overexpressing lines (OE2 and OE6) in a Nip background (Figure 6A,B). The *SbERF027*-overexpressing lines had significantly higher survival rates than WT plants under cold stress, and no differences in survival rates were observed between these plants under normal conditions (Figure 6C). Moreover, the chlorophyll content can be used to evaluate the extent of cold injury to plants [16,54], which was further determined in transgenic lines and WT plants. The chlorophyll content of *SbERF027*-overexpressing lines was significantly higher than that of WT plants after cold treatment, but no difference in the chlorophyll content was observed under normal conditions (Figure 6D). To further characterize the cold tolerance of *SbERF027*-overexpressing plants, we detected the content of EL, Pro, and MDA before and after cold treatment. The EL and MDA content of *SbERF027*-overexpressing lines was significantly lower than that of WT plants under cold treatment, and the Pro content was significantly higher in *SbERF027*-overexpressing lines than in WT plants (Figure 6E–G); however, no significant differences between them were observed under normal conditions. To further clarify the function of *SbERF027*, two gene-silenced lines of sorghum (*sberf027*-1, *sberf027*-2) were obtained by VIGS. The survival rate and content of Pro and chlorophyll were significantly lower in gene-silenced plants than in control plants after cold stress, and the EL and MDA content was significantly higher in gene-silenced plants than in control plants (Figure 7A–G). The above results indicate that *SbERF027* positively regulates cold tolerance at the seedling stage in sorghum, but the molecular mechanism underlying its regulatory effects on cold tolerance requires further study.

### 2.8. Analysis of SbERF027 Expression Patterns

We analyzed the expression patterns of *SbERF027* by qRT-PCR and subcellular localization analysis. The results of the tissue expression analysis showed that *SbERF027* was expressed in the seedlings, roots, stems, leaves, sheaths, and panicles, and it was highly expressed in the seedlings and panicles (Figure 5B). The expression of *SbERF027* was induced by cold stress, and it was most highly expressed at 2 h and 8 h during cold treatment in SZ7 and Z-5, respectively. Furthermore, the cold-induced expression level of *SbERF027* was higher in SZ7 than in Z-5 (Figure 5C). This result was consistent with the results of the phenotypic evaluation, indicating that *SbERF027* positively regulated cold tolerance at the seedling stage in sorghum. Additionally, subcellular localization analysis showed that the green fluorescence signal of SbERF027 was detected in the nucleus under both normal and cold stress conditions (Figure 5D), suggesting that SbERF027 mainly accumulates in the nucleus under both normal and cold stress conditions; the localization of SbERF027 was not altered by cold.

### 2.9. SNP-911 on the Promoter of SbERF027 Was Responsible for Transcriptional Differences

**Figure 8:**
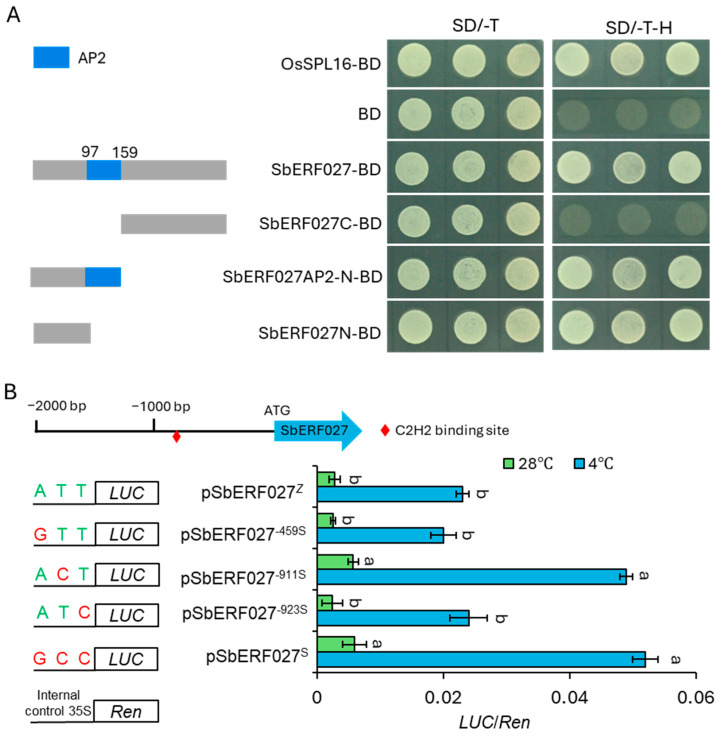
*SbERF027* positively regulates cold tolerance in sorghum. (**A**) Transactivation assays of SbERF027-truncated mutants in yeast cells transformed with different constructs on different selective media. SD/-T and SD/-T-H mean the SD/-Trp medium and SD/-Trp-His medium, respectively. The OsSPL16-BD construct and empty BD were used as positive and negative controls, respectively. (**B**) Transient expression assay of promoter activity under normal conditions (blue) and cold treatment (green) in wheat protoplasts by using *SbActin1* as internal reference. Left, constructs with site-directed mutations at the three single SNPs in the promoter region. Right, relative LUC/Ren values. LUC, luciferase; Ren, Renilla. Error bars represent SD (n = 3) (Tukey’s test). Significant differences are represented by different lowercase letters above the bars.

To determine whether the transcriptional activity of *SbERF027* was similar to that of other TF members, transcriptional activation experiments were performed. The results showed that transcriptional activation activity was observed in the entire gene region, and the N-terminus was essential for the transcriptional activation of *SbERF027* (Figure 8A). Additionally, sequence comparison revealed that no differences in the SbERF027 coding sequence (CDS) were detected between SZ7 and Z-5, while several SNPs (SNP-459, SNP-911, and SNP-923) were observed on the promoter between SZ7 and Z-5. Additionally, SNP-911 and SNP-923 were located within a *cis*-regulatory element with unknown function based on the PlantCARE database, and SNP-911 is a potential binding site for C2H2 subfamily TFs according to the PlantDTBD database (Figure S12, Table S22). To determine the functional SNPs (FNPs) underlying cold tolerance, the transcriptional activity of various *SbERF027* promoter fragments in protoplasts was measured using a dual-luciferase (dual-LUC) assay, and each specific SNP from the *SbERF027* promoter of SZ7 (p*SbERF027*^S^) was individually introduced into the *SbERF027* promoter from Z-5 (p*SbERF027*^Z^). The relative LUC activity derived from p*SbERF027*^S^ was much higher than that obtained with p*SbERF027*^Z^, and this was consistent with the previously measured *SbERF027* transcript levels (Figure 5C). We then tested each SNP individually by replacing the nucleotide from Z-5 with the equivalent nucleotide from SZ7. The LUC activity of only the SNP-911 promoter variant was comparable to that of p*SbERF027*^S^, while the SNP-923 and SNP-459 variants did not result in significant changes in LUC activity relative to p*SbERF027*^Z^ (Figure 8B).

To determine whether low temperature affects the promoter activity of *SbERF027*, the LUC activity was measured according to a previous report [5]. The promoter activity was significantly increased under cold stress in both the *SbERF027*^S^ and *SbERF027*^Z^, and the *SbERF027*^S^ promoter activity was prominently higher than that of the *SbERF027*^Z^ (Figure 8B). These results suggested that SNP-911 in the promoter of *SbERF027* was the FNP responsible for the differences in transcriptional output between the *SbERF027*^S^ and *SbERF027*^Z^ alleles, and cold stress promotes the transcriptional activity of *SbERF027*.

## 3. Discussion 

### 3.1. Discovery of Genes for Cold Tolerance in Sorghum Seedlings

Plants are sessile organisms that are constantly exposed to various types of abiotic stress, such as drought, cold, and salinity stress [55,56,57,58,59]. Exposure to low temperatures, especially in the early stage of plant growth, can affect the growth, development, and geographical distribution of plants, and cold tolerance is required for sorghum with high productivity and quality to be cultivated in cooler areas [48]. Although cold tolerance is a complex quantitative trait and phenotypes are easily affected by environmental factors, several methods have been used to determine the cold tolerance of sorghum and other tropical crop plants such as rice and maize during the early stage of growth [6,60,61,62,63,64,65,66]. Several QTLs or hotpots have been identified through forward and reverse genetic approaches, such as bi-parental mapping, GWASs, GBS, RNA-seq, and metabolomic analyses [2,17,18,19,20,21,22,23,24,25]. In this study, we identified an AP2/ERF transcription factor family gene *SbERF027* for cold tolerance via RNA-seq by sampling leaves of sorghum seedlings before and after exposure to cold stress. We identified the metabolic pathways and molecular mechanisms involved in cold stress through comparative analysis, including GO, KEGG, and TF analysis based on the DEGs obtained in the RNA-seq data of the two sorghum varieties differing in cold tolerance.

### 3.2. Gene Function and Regulatory Pathways Under Cold Stress

The susceptibility or tolerance of plants to low temperatures largely depends on the activation of genes involved in the response to cold stress in the MF, CC, and BP categories [52,67,68]. In our study, a total of 10,663 GO terms were identified through GO enrichment analysis in both varieties, and 717 of them met the significant enrichment criteria (Figure S4). Further comparative analysis revealed one and five GO terms that were shared among the significantly enriched terms in Z-5 and SZ7, respectively, and the GO term “Response to cold” was detected in SZ7, but not in Z-5, indicating that the response to external cold stress differed among accessions differing in cold resistance; cold-resistant varieties might show a greater diversity of responses to cold stress than cold-sensitive materials (Figure S7). Previous studies indicate that genes enriched in “Response to cold” play a crucial role in regulating cold tolerance in crops such as rice [5]. A total of 86 DEGs were enriched in “Response to cold” in SZ7, and the number of genes enriched in “Response to cold” increased gradually with the duration of cold treatment. Only one gene, *SORBI_3003G425500*, encoding a probable calcium-binding protein (CML10) was commonly observed in the three SZ7 comparison groups (Figure 3A,B, Table S17). Previous studies have shown that the calmodulin-related calcium sensor protein plays a role in responses to abiotic stress, such as cold, drought, and salinity stress, in diverse plants including rice, wheat, and bean [5,58,69,70,71,72], suggesting that this gene may play an important role in the response to low temperature or other abiotic stresses; these findings have implications for future studies aimed at identifying important candidate genes involved in responses to abiotic stress.

Sorghum is more susceptible to cold stress than other monocotyledonous species in the early spring; it is thus critically important for revealing the internal genetic mechanism and regulatory pathways involved in cold stress at the seedling stage. In this study, 5784 DEGs were enriched in KEGG pathways in both accessions, which is consistent with the results of the GO analysis; the number of enriched KEGG pathways in the two accessions gradually increased as the duration of low-temperature stress extended, and the number of enriched KEGG pathways was significantly higher for the up-regulated genes than for the down-regulated genes in most comparison groups, indicating that plants might adapt to changes in environmental factors by regulating gene expression levels in response to abiotic stresses, such as low temperature. The number of KEGG pathways enriched in cold-sensitive varieties and cold-tolerant varieties was highest in Z_T3 and S_T2, respectively, indicating that the responses to cold stress of these varieties are temporally variable, especially among materials differing in cold resistance (Figure S8). Among significantly enriched KEGG pathways, several KEGG pathways, such as Photosynthesis, Porphyrin metabolism, Carbon metabolism, and Biosynthesis of various plant secondary metabolites, were previously reported to be involved in cold stress and play a crucial role in responses to diverse types of abiotic stress in plants (Figures S9 and S10) [48,73,74]. Moreover, four genes were commonly detected in the two materials through comparative analysis of DEGs from KEGG pathways enriched in both materials, and one gene, *SORBI_3003G425500*, was the same as that identified in a previous analysis (Figure S8), indicating that *SORBI_3003G425500* may be an important candidate gene for cold tolerance at the seedling stage in sorghum; however, additional studies are needed to clarify the specific mechanism of cold tolerance.

### 3.3. AP2/ERF TFs Play a Pivotal Role in the Cold Stress Response in Plants

TFs are responsible for positively or negatively regulating the expression of target genes by binding to specific motifs on the promoter, which has a direct effect on the expression of target genes [47]. In this study, a total of 746 differentially expressed TFs from more than 30 TF families were detected in both genotypes (Figure 4A and Figure S11), and many TF families, such as MYB, ERF, bHLH, NAC, bZIP, WRKY, C2H2, and NF TFs, have been shown to play essential roles in the response to abiotic stress in various plants [46,49,50,51,52,53,75]. AP2/ERF TFs play a crucial role in the response to various abiotic stresses, and AP2/ERF family genes have been shown to play a role in abiotic stresses such as cold, drought, and salinity stress in various crops, including rice, maize, saccharum, and foxtail millet [32,33,34,35,36,37,38,39,40,41,42]. Phosphorylated OsERF52 directly up-regulates the expression of C-repeat binding factor (CBF) genes to positively modulate cold tolerance in rice [39], indicating that AP2/ERF members in other crops may also regulate cold tolerance through the CBF pathway. The CBF/DREB transcriptional regulatory cascade is the most well-studied cold-signaling pathway in plants. Under cold conditions, CBF genes can respond rapidly to cold stress and activate the expression of downstream genes that protect plants from cold injury; they eventually modulate cold tolerance through the ICE1-CBFsCOR regulatory network in plants [39,76,77,78]. In sorghum, the expression of SbCBF6 is induced and highly expressed in cold-tolerant sorghum under cold conditions [1,79]. Additionally, 158 ERF family genes, including 52 DREBs and 106 ERF subfamily proteins, have been identified in sorghum [43], and the function of AP2/ERF family proteins in the regulation of cold tolerance, as well as the genetic and molecular basis of their effects, has not been well studied in sorghum.

### 3.4. Possible Involved Pathways of SbERF027 in Response to Cold Stress in Sorghum

In the present study, we identified an AP2/ERF gene, *SbERF027*, conferring cold tolerance via mediating its expression in the sorghum seedings. Although the genetic mechanism of *SbERF027* involved in the regulation of cold tolerance in sorghum remains unclear, the molecular mechanism responding to cold tolerance of its orthologous genes has been reported. For example, the orthologous genes *OsDREB1A*/*OsCBF3* have been reported to be involved in the regulation of cold tolerance in rice. *OsDREB1A* positively regulates cold tolerance by regulating the expression of cyclic nucleotide-gated channel protein OsCNGC9, which in turn regulates chilling tolerance by mediating cytoplasmic calcium elevation [35]. *OsCBF3* is also involved in the regulation of cold tolerance through the OsSAPK6-IPA1-OsCBF3 module in rice. OsSAPK6 phosphorylates the IPA1 protein, leading to its accumulation and the subsequent activation of downstream OsCBF3 expression, thereby enhancing the cold stress resistance of rice [37]. In *A. thaliana*, BIN2 mediated the attenuation process of CBFs via phosphorylating ICE1 under prolonged low-temperature stress, facilitating the interaction between HOS1 (Osmotically Responsive Gene1) and ICE1 and resulting in the degradation of ICE1 [80]. BZR1 (Brassinazole-Resistant 1) acts as an upstream regulator of *CBF1* and *CBF2*, directly regulating their expression to increase cold stress tolerance in *A. thaliana* [81]. It has been reported that DELLAs contribute to the cold induction of *CBF1*, *CBF2*, and *CBF3* via JA signaling. In addition, *CBF3* improves DELLA accumulation by suppressing GA biosynthesis [82]. Low temperature induced the activation of *ICE1* and *ICE2* by endogenous JA, leading to the activation of the CBF/DREB1 transcriptional cascade [83]. Previous findings have also demonstrated that the expression of CBFs is also influenced by several factors such as exogenous ABA, circadian clock, eugenol, and light conditions under cold stress [84,85]. In *S. melongena*, CBFs were strongly, rapidly, and transiently induced by exogenous ABA, indicating that *SmCBFs* might be affected by plant response to ABA [86]. Taken together, orthologous or subfamily genes of *SbERF027* have been reported to participate in plant response to cold stress through different regulatory networks, including cytoplasmic calcium elevation, post-translational modification of phosphorylation and ubiquitination, and JA and ABA biosynthesis pathways, which will provide a reference for dissecting the genetic mechanisms of *SbERF027* under cold stress in sorghum.

## 4. Conclusions

In this study, we functionally identified the AP2/ERF gene *SbERF027* in sorghum through a comparative analysis. *SbERF027* was more highly expressed in seedlings and panicles, and the cold-induced expression level of *SbERF027* was significantly higher in SZ7 than in Z-5. *SbERF027* was located in the nucleus before and after cold stress. Additionally, *SbERF027* was identified as a positive regulator to improve the tolerance of low-temperature stress in sorghum, and SNP-911 of the promoter was essential for enhancing cold tolerance by mediating *SbERF027* expression output. However, additional studies of the mechanisms underlying the regulation of cold tolerance by *SbERF027* are needed.

## 5. Materials and Method

### 5.1. Plant Culture and Phenotypic Evaluation

Two sorghum varieties, cold-tolerant Suiza7 (SZ7) and cold-sensitive Zhong-5 (Z-5) from northern China, were cultured in soil/hydroponic pots in plant growth climate boxes (ZRX-460B-LED, Ningbo, China), and seedlings at trefoil stage were used as materials for phenotypic evaluation and determination of physiological indices under normal conditions. Briefly, seeds with similar sizes were screened for germination at 30 °C for 2–3 days, and then the seeds with a consistent bud length were selected and transferred to earth pots or hydroponic boxes for cultivation for 5–8 days (there are different growth rates between sorghum and rice) until they grew to the trefoil stage. Leaves at the seedling stage were collected for transcriptome analysis before (0 h) and 2 h, 8 h, and 16 h after cold treatment. The leaves of tissue culture seedlings were stored at −80 °C. Three biological replicates of each sample were collected.

Phenotypes were evaluated using the following method: cold treatment was conducted with seedings at the trefoil stage in a plant growth climate box at 4 °C (14 h day with 13,000 LUX/10 h night with 0 LUX) for 7 d, and the seedling survival rate and various physiological indices were measured after recovery for 7 days under normal conditions (25 °C).

### 5.2. Transcriptome Analysis

The leaves of two varieties before and after cold treatment (4 °C) were used for RNA-seq. The total RNA of leaves was extracted using the mirVana miRNA Isolation Kit (Ambion-1561, Austin, TX, USA). RNA concentration, purity, and integrity were evaluated using an Agilent 2100 Bioanalyzer (Agilent Technologies, Santa Clara, CA, USA). The libraries were constructed using a TruSeq Stranded mRNA LTSample Prep Kit (Illumina, San Diego, CA, USA). The Illumina sequencing platform (Illumina HiSeq) was used to sequence libraries. Gene expression levels were measured using Fragments Per Kilobase of transcript per Million mapped reads (FPKM) values. Gene Ontology (GO) and Kyoto Encyclopedia of Genes and Genomes (KEGG) enrichment analyses were performed on the differentially expressed genes (DEGs) to describe their functions and pathways using the online tool (https://cloud.oebiotech.cn/task/). The significance of the enrichment of DEGs in each GO and KEGG pathway term was determined using R software (vision 4.1.0) with the clusterProfiler package [87].

### 5.3. Plasmid Construction and Genetic Transformation

The coding sequence of SbERF027 was amplified without the stop codon using the cDNA of SZ7 as a template and cloned into the empty Super1300-GFP and pMC1307 vectors to generate the fusion construct for subcellular localization (35S: SbERF027-GFP) and overexpression transgenic plants (35S: SbERF027-Flag), respectively. To generate the vector of pGreenII0800-LUC-pSbERF027^S^ and pGreenII0800-LUC-pSbERF027^Z^, a 2 kb fragment of the SbERF027 promoter from SZ7 and Z-5 was amplified and cloned into pGreenII0800-LUC. Mutated forms of the SbERF027 promoter (pSbERF027-459S, pSbERF027-911S, and pSbERF027-923S) were obtained by site-directed mutagenesis using the pGreenII0800-LUC^Z^ plasmid as a template and then cloned into empty pGreenII0800-LUC to generate pGreenII0800-LUC-pSbERF027-459S, pGreenII0800-LUCpSbERF027-911S, and pGreenII0800-LUC-pSbERF027-923S, respectively. All vectors were constructed by recombination cloning (Trelief^®^ SoSoo Cloning Kit, Beijing, China), and primer information is shown in Table S23.

To generate the overexpression transgenic plants, *Agrobacterium tumefaciens* (EHA105) carrying overexpression plasmids was incubated at 28 °C for 3–4 days and infiltrated into Nipponbare (Nip); the transgenic seedlings were obtained after screening on culture medium [88]. Homozygous transgenic plants were selected for hygromycin resistance and used in subsequent experiments.

Sorghum plants with *SbERF027* silenced via VIGS were generated following the methods described in a previous study [89]. An approximately 450 bp fragment of *SbERF027* was cloned and inserted into the pTRV2 vector with tobacco rattle virus (TRV) to obtain the pTRV2-SbERF027 vector. The pTRV2 and pTRV2-SbERF027 vectors were transformed into *Agrobacterium tumefaciens* strain GV3101 using the thermal stimulation method (The plasmid was first incubated with ice for 30 min, then rapidly frozen with liquid nitrogen for 5 min, and incubated at 37 °C for 5 min, and rapidly incubated with ice for 2 min) for genetic modification. The germinating seeds of sorghum with approximately 1 cm emerging shoots were infiltrated with the bacterial solutions, and the washed seedlings with ddH_2_O were placed in a controlled growth climate box (ZRX-460B-LED, Ningbo, China) for two weeks. Sorghum leaves at the trefoil stage were sampled for the analysis of transcript levels, and seedlings with low *SbERF027* expression were selected for further analysis. The primers used for plasmid construction in this study are listed in Table S23.

### 5.4. Expression Patterns and Subcellular Localization

To analyze the expression of cold-induced candidate genes, leaves of the two cultivars at the trefoil stage were collected after 0 h, 2 h, 8 h, 16 h, 2 d, 4 d, and 6 d of cold treatment at 4 °C. Various tissues, including roots, stems, leaves, sheaths, and panicles, were harvested from SZ7 under normal conditions to detect expression patterns. Total RNA was extracted using RNAiso Plus, and reverse transcription was performed using M-MLV reverse transcriptase (Takara, Kyodo, Japan) to detect the expression patterns of candidate genes. The *SbActin1* gene was used as an internal reference to perform qRT-PCR analysis on an ABI 7500 instrument (Applied Biosystems, Foster City, CA, USA). Three biological replicates were performed for all expression analyses. Student’s *t*-test was performed to analyze the data. All primers used for gene expression analysis are listed in Table S23.

Subcellular localization of SbERF027 was conducted following a previous report [90] with minor modifications. Wheat seedlings grown for 10 days under normal conditions were used to extract protoplasts. They were first cut and placed into the enzyme solution, vacuumed at 40 Pa for 20–30 min, and shaken on a 50 rpm shaker under dark and room temperature conditions for 3 h. The mixture of chopped seedlings and solution was filtrated and collected; it was then centrifuged at 250× *g* for 5 min to remove the supernatant. Protoplasts were re-suspended and extracted by adding W5 buffer; they were then centrifuged at 250× *g* for 5 min, and W5 buffer was added again to obtain the protoplasts. The subcellular localization of SbERF027 was detected via PEG-mediated transient transfection in wheat protoplasts with a 35S:SbERF027-GFP fusion construct driving the expression of SbERF027 fused to the GFP under control of the 35S promoter. The coding sequence of SbERF027 was amplified without the stop codon with the DNA polymerase (Tks GlexTM, Takara, Japan), cloned to generate the 35S:SbERF027-GFP construct, and transfected into wheat protoplasts with 35S:SbERF027-GFP. The vectors of OsSPL16-mCherry as a positive control were cotransfected into protoplasts with 35S:SbERF027-GFP. The empty GFP vector was used as a negative control; samples were cultured at 28 °C for 16–18 h in the dark or further treated at 4 °C for 20 min based on the above culture process to detect the subcellular localization of SbERF027 under cold stress. The subcellular localization of SbERF027 was observed with a confocal microscope. All primers used for subcellular localization analysis are listed in Table S23.

### 5.5. Determination of Physiological Indices

The chlorophyll content was measured following the method described in a previous study [91]. The leaves of sorghum plants were collected before and after cold treatment. Next, 95% ethanol solution was used to extract chlorophyll, and the mixtures were left to stand for 2–3 h under dark conditions. The supernatants were obtained via centrifugation, which was used to measure the absorption values at 646 and 663 nm using an ultraviolet spectrophotometer. The formula for calculating the chlorophyll content was as follows: ‘Total chlorophyll content (mg/g) = (12.21 × A663–2.81 × A646 + 20.13 × A646–5.03 × A663) × V/1000 × W’, where W (g) and V (mL) represent the weight of sorghum leaves ground with liquid nitrogen and the total volume of the added extract, respectively.

The proline (Pro) content was determined using a kit (Grace, Suzhou, Jiangsu Province, China) following the manufacturer’s protocol. The electrolyte leakage (EL) was determined based on the method described in a previous study with minor modifications [92]. Leaves at the seedling stage were collected before and after cold treatment, placed into tubes with 40 mL of double-distilled water, and shaken for 4–5 h. The initial conductivities of the blank control (A0) and tubes with leaf segments (A1) were measured using a Mettler-Toledo instrument with the model of the electrode constant 0.996 at 25 °C (Shanghai, China). The sample conductivities (A2) were measured with the tubes after they were incubated in boiling water for 15–20 min and cooled to room temperature. The ratio of A1−A0 to A2−A0 was used to calculate the relative electrolyte leakage. Three biological replicates were performed for each sample, and Tukey’s post hoc tests were performed to analyze the data.

### 5.6. Transcriptional Activity Assays

Transcriptional activity of the *SbERF027* promoter was performed according to the method described in a previous study [55]. Through the transient transformation, several fusion constructs of mutated pSbERF027::LUC were cotransfected into protoplasts using 35S::Ren (*Renilla* LUC) as an internal control. Protoplasts were cultured at 28 °C for 16–18 h in the dark to detect transcriptional activity under normal conditions or were treated at 4 °C for 20 min based on the above culture process to measure transcriptional activity under cold stress. The quick protocol (Dual Luciferase Reporter Assay System manual) was used to prepare the 1 × PLA, LAR II, and Stop & Glo reagents. The cultured protoplasts were then collected by centrifugation, and 100 μL of lysate 1 × PLA was added; 50 μL of LAR II was then added after vortexing for 10 s and quickly mixed to determine firefly LUC activity. Ren activity was immediately detected after adding 50 μL of Stop & Glo Reagent. The activity ratio of LUC to Ren was used to calculate the relative promoter activity. Three biological replicates were performed for each construct. Tukey’s post hoc tests were performed to analyze the data.

### 5.7. Transactivation Assays of SbERF027

To determine the transcriptional activity of *SbERF027*, the full-length and truncated coding sequences of *SBERF027* were cloned into pGBKT7. The OsSPL16-BD construct and empty BD were used as positive and negative controls, respectively. All the above vectors were transformed into the yeast (AH109) cells, and the recombinant yeasts were screened and cultured on SD/-Trp plates at 30 °C. Then recombinant yeasts normalized with OD600 = 0.6. μL aliquots of 10-fold serial diluted culture were spotted on SD/-Trp and SD/-Trp-His agar plates for comparing the growth of recombinant yeasts.

## Data Availability

The original contributions presented in the study are included in the article and Supplementary Material; further inquiries can be directed to the corresponding author.

## Supplementary Materials

The following supporting information can be downloaded at https://www.mdpi.com/article/10.3390/plants14060879/s1. Figure S1: Evaluation of cold tolerance for two sorghum varieties via earth culture. Figure S2: Clustering analysis of samples used in this study. Figure S3: Validation of RNA-seq results through qRT-PCR. Figure S4: Statistic analysis of GO terms for two varieties at different time intervals. Figure S5: Functional annotation analysis of DEGs in Z-5. Figure S6: Functional annotation analysis of DEGs in SZ7. Figure S7: Comparative analysis of GO terms enriched in Z-5 and SZ7. Figure S8: Integrated comparison of KEGG pathways for two varieties at different time intervals. Figure S9: KEGG pathways significantly enriched in Z-5 at different time intervals. Figure S10: KEGG pathways significantly enriched in SZ7 at different time intervals. Figure S11: Statistics of TFs families and numbers significantly enriched both in Z-5 and SZ7. Figure S12: Alignment on promoter 2 k genome sequences of *SbERF027* between SZ7 and Z-5. Table S1: Summary of sample quality after filtering. Table S2: Summary of sample reads alignment. Table S3: Summary for regional distribution of the sample reads on the genome. Table S4: DEGs of Category1_Z-5_T1 vs Z-5_T0. Table S5: DEGs of Category2_Z-5_T2 vs Z-5_T0. Table S6: DEGs of Category3_Z-5_T3 vs Z-5_T0. Table S7: DEGs of Category1_SZ7_T1 vs SZ7_T0. Table S8: DEGs of Category2_SZ7_T2 vs SZ7_T0. Table S9: DEGs of Category3_SZ7_T3 vs SZ7_T0. Table S10: GO terms of Category1_Z-5_T1 vs Z-5_T0. Table S11: GO terms of Category2_Z-5_T2 vs Z-5_T0. Table S12: GO terms of Category2_Z-5_T3 vs Z-5_T0. Table S13: GO terms of Category1_SZ7_T1 vs SZ7_T0. Table S14: GO terms of Category1_SZ7_T2 vs SZ7_T0. Table S15: GO terms of Category1_SZ7_T3 vs SZ7_T0. Table S16: GO information commonly detected at different time points in Z-5 and SZ7. Table S17: DEGs enriched in terms of Category “Response to cold” in Z-5 and SZ7. Table S18: KEGG pathway of signifcantly enriched in Z-5 at different time intervals. Table S19: KEGG pathway of signifcantly enriched in SZ7 at different time intervals. Table S20: Up and down regulation of DEG numbers significantly enriched in KEGG pathways both in Z-5 and SZ7 at different time intervals. Table S21: Log2FC data of 12 TFs in different combinations. Table S22: Motifs pridiction in promoter of SZ7 and Z-5. Table S23: Primers used in this study.
