# Supplementary material for: Transcriptome Analysis Reveals the Pivotal Genes and Regulation Pathways Under Cold Stress and Identifies SbERF027, an AP2/ERF Gene That Confers Cold Tolerance in Sorghum
Source: Plants (Basel). 2025 Mar 11;14(6):879. doi: 10.3390/plants14060879 (PMC11944419; doi:10.3390/plants14060879)
Supplement: Supplementary file 1 [file plants-14-00879-s001.zip › plants-3372981-supplementary figures.pdf]

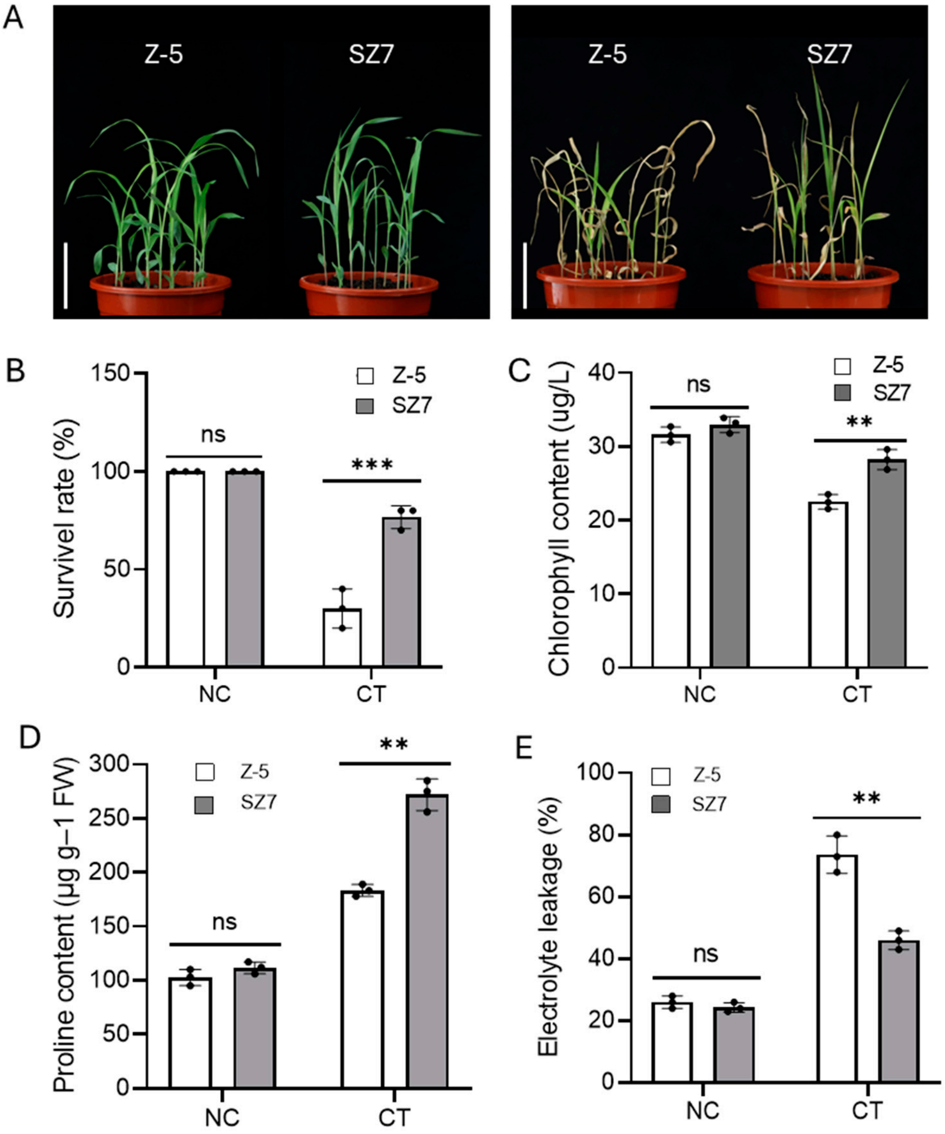

Figure S1 Evaluation of cold tolerance for two sorghum varieties via earth culture. (A–B) Seedling phenotype (A) and survival rate evaluated of SZ7 and Z-5 under normal and cold stress conditions. Scale bars, 4 cm. (C–E) The content of chlorophyll (C), Pro (D) and EL (E). Error bars represent standard deviation (SD). Statistical significance was determined by two-tailed Student's *t*-test: **, P < 0.01, *** P < 0.001. no significance.

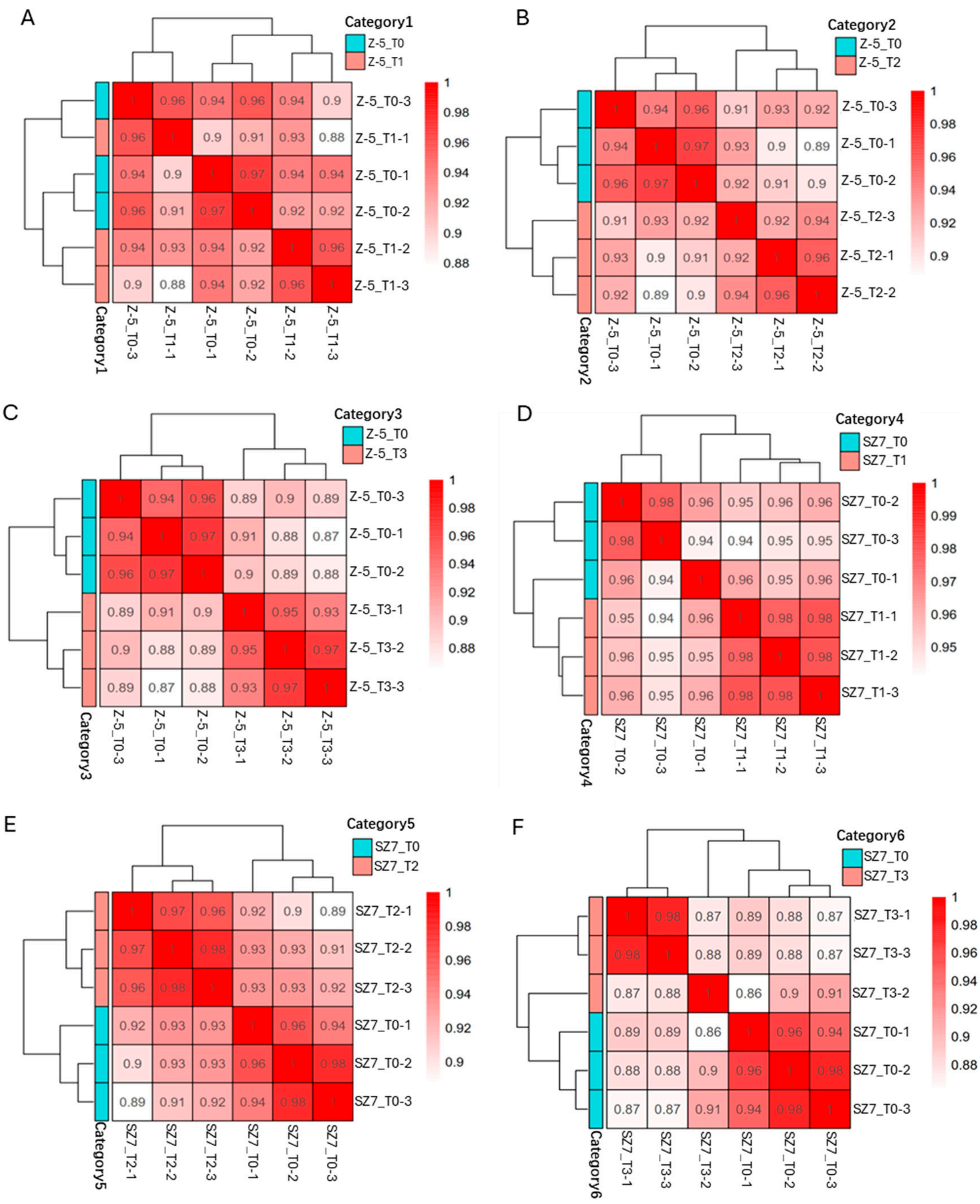

Figure S2 Clustering analysis of samples used in this study. (A–F) Unsupervised hierarchical clustering of samples for Z-5 at 0 h (T0), 2 h (T1), 8 h (T2), and 16 h (T3), respectively. Red indicates a high correlation between the two samples.

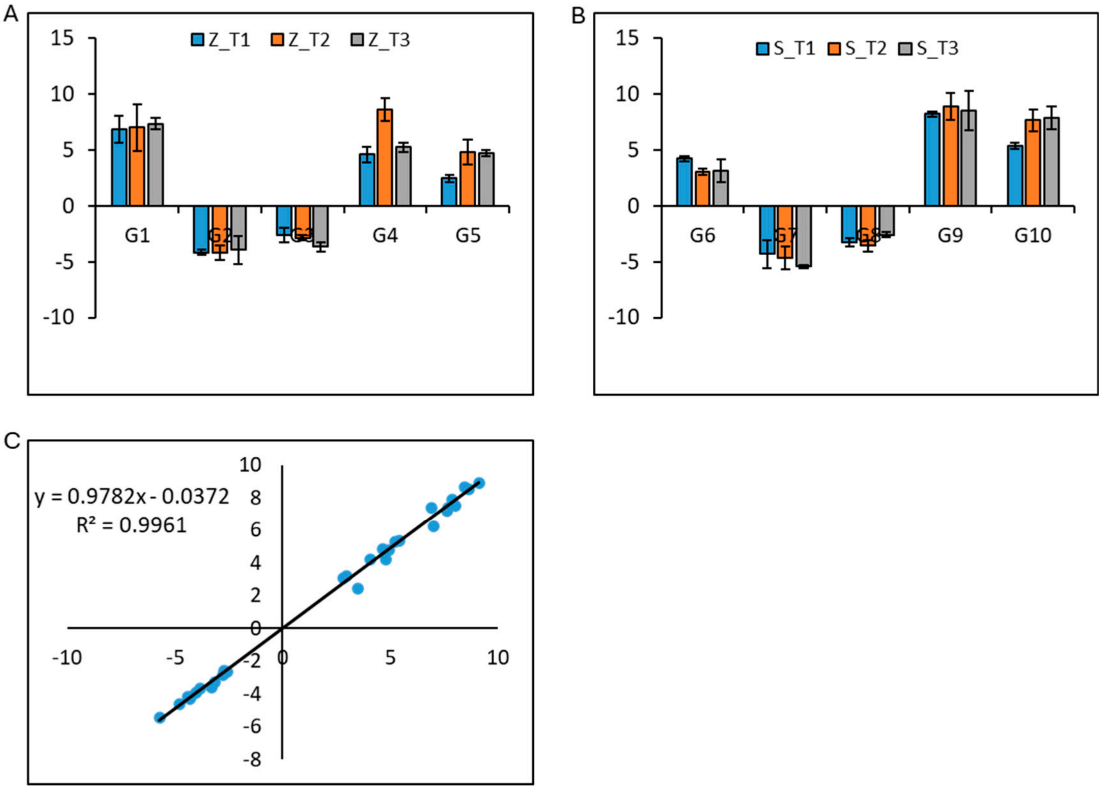

Figure S3 Validation of RNA-seq results through qRT-PCR. (A–B) expression level of 10 DEGs randomly selected from combinations of ECZ (A) and ECS (B) via qRT-PCR method, respectively.G1: *SORBI_3005G212700*, G2: *SORBI_3003G142300*, G3: *SORBI_3004G025900*, G4: *SORBI_3003G064200*, G5: *SORBI_3002G157050*, G6: *SORBI_3001G074400*, G7: *SORBI_3010G104600*, G8: *SORBI_3002G180100*, G9: *SORBI_3003G284400*, G10: *SORBI_3004G229300*. B. Correlation analysis of DEGs between qPCR analysis and RNA-seq data.

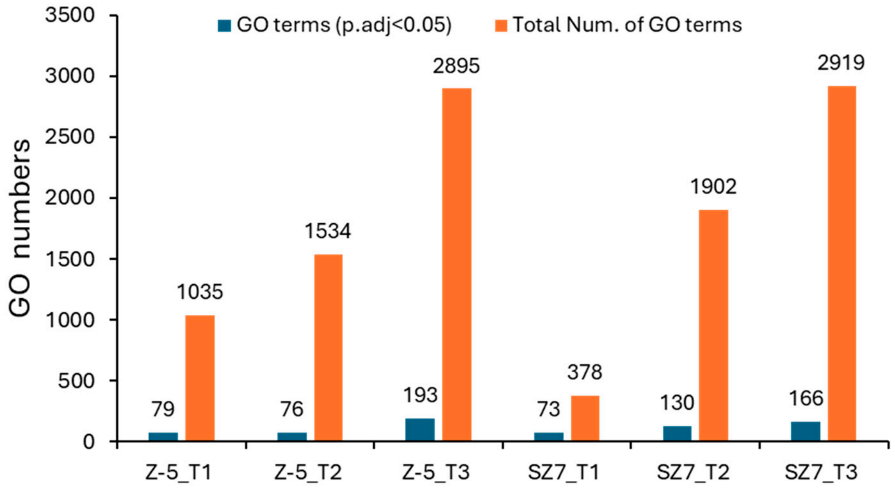

Figure S4 Statistic analysis of GO terms for two varieties at different time intervals.

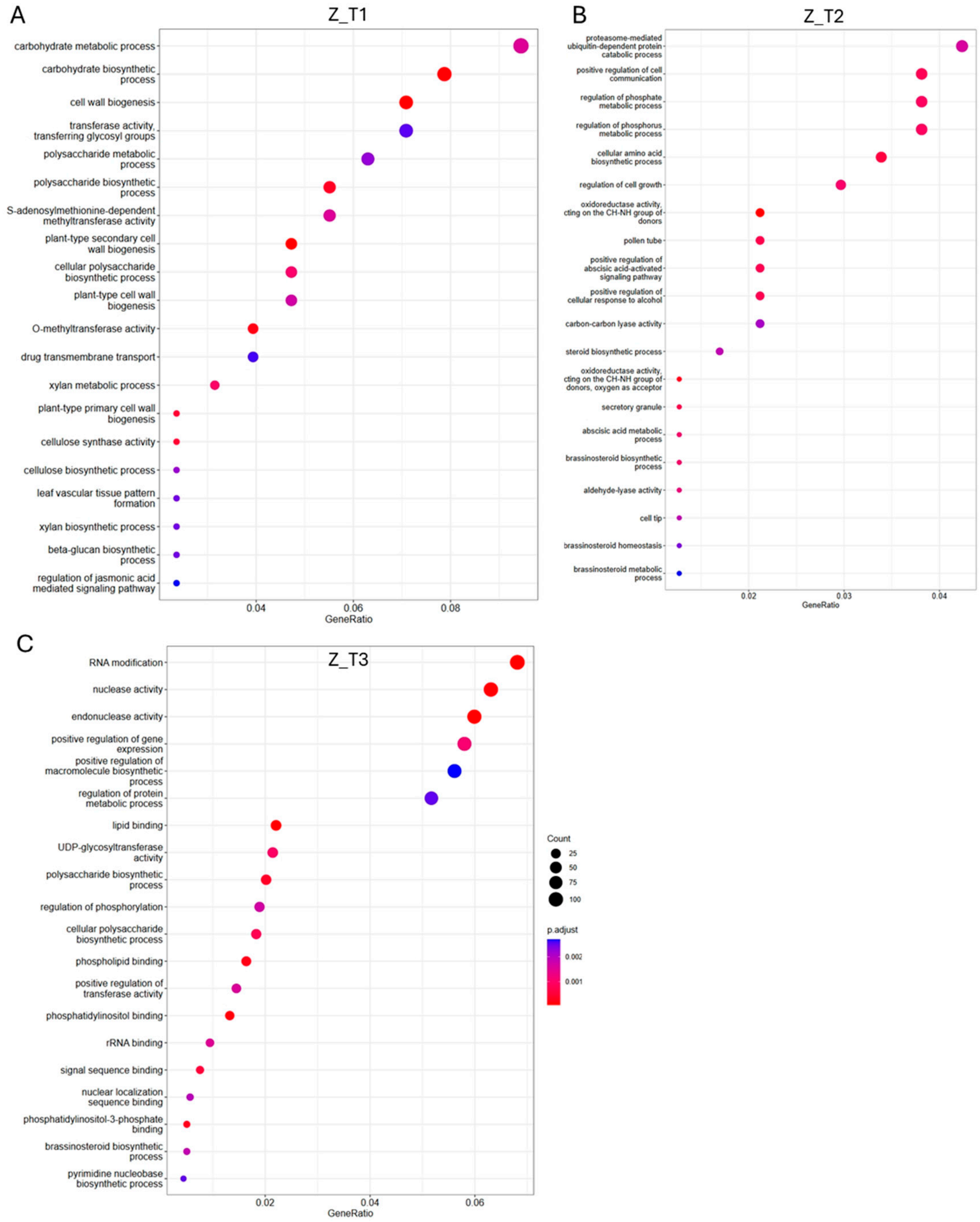

Figure S5 Functional annotation analysis of DEGs in Z-5. (A–C) Histogram displaying top 20 significantly enriched GO terms from Z_T1 (A), Z_T2 (B), Z_T3 (C), respectively.

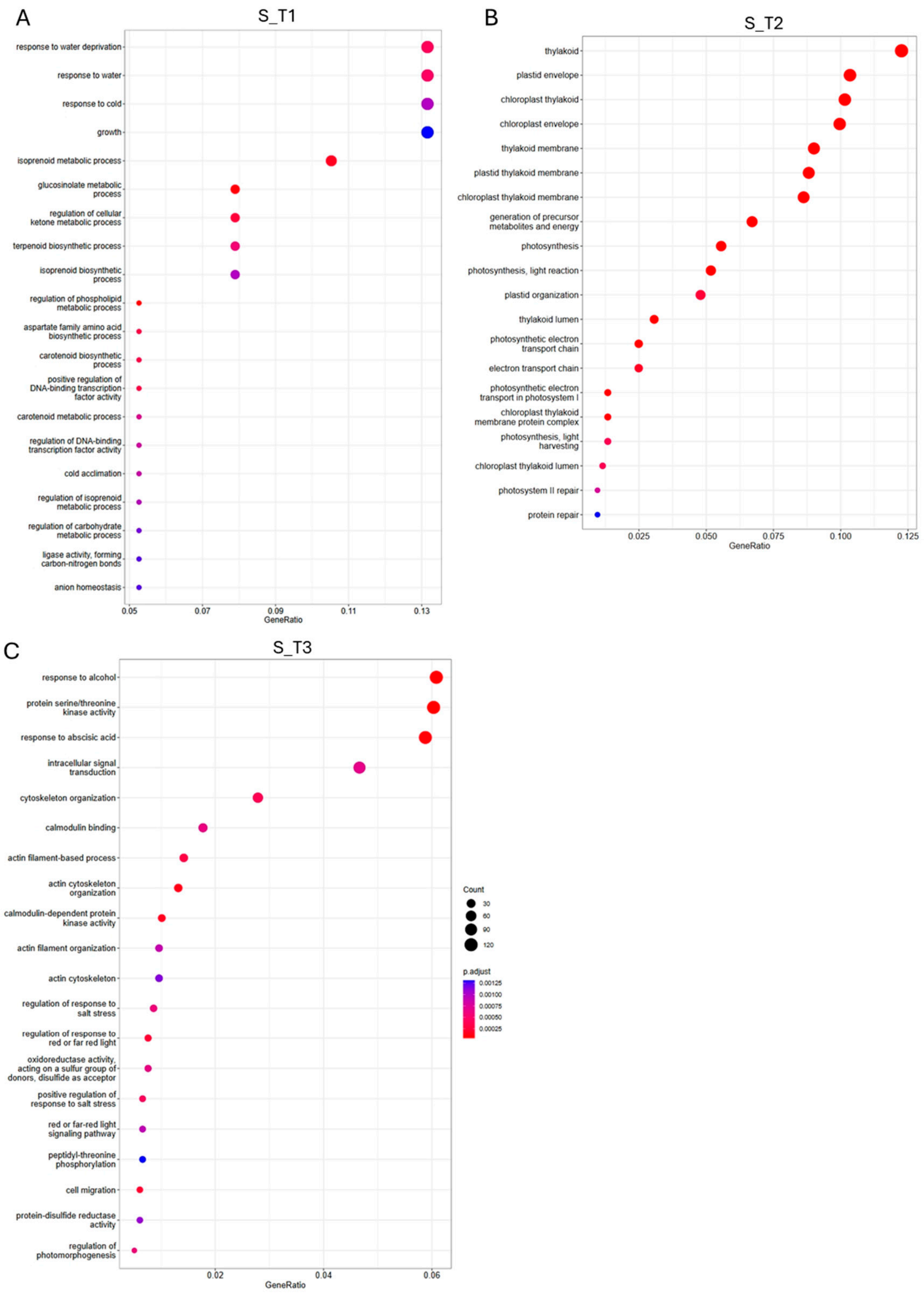

Figure S6 Functional annotation analysis of DEGs in SZ7. (A–C) Histogram displaying top 20 significantly enriched GO terms from S_T1 (A), S_T2 (B), S_T3 (C), respectively.

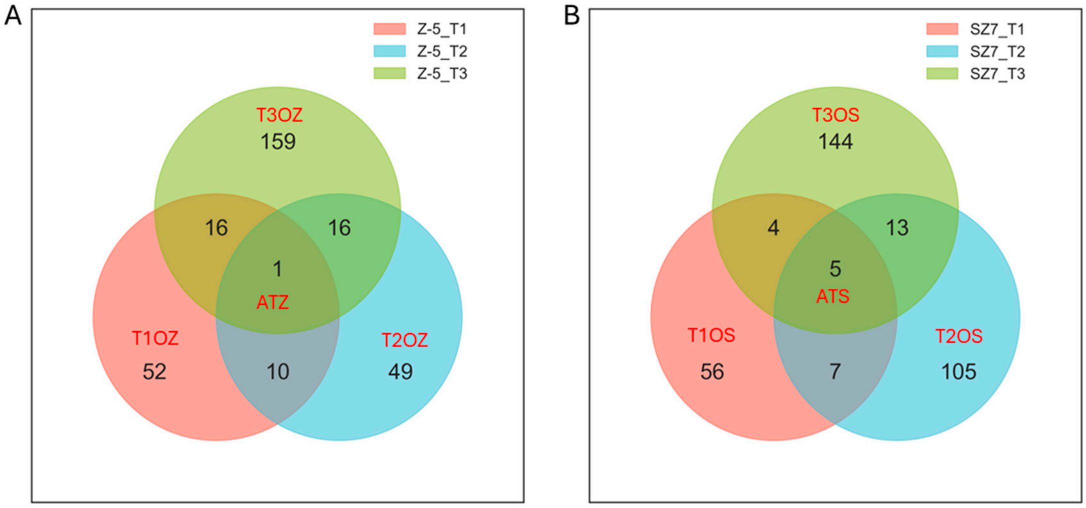

Figure S7 Comparative analysis of GO terms enriched in Z-5 and SZ7. (A–B) Venn diagram of Z-5 (A) and SZ7 (B) at different time intervals.

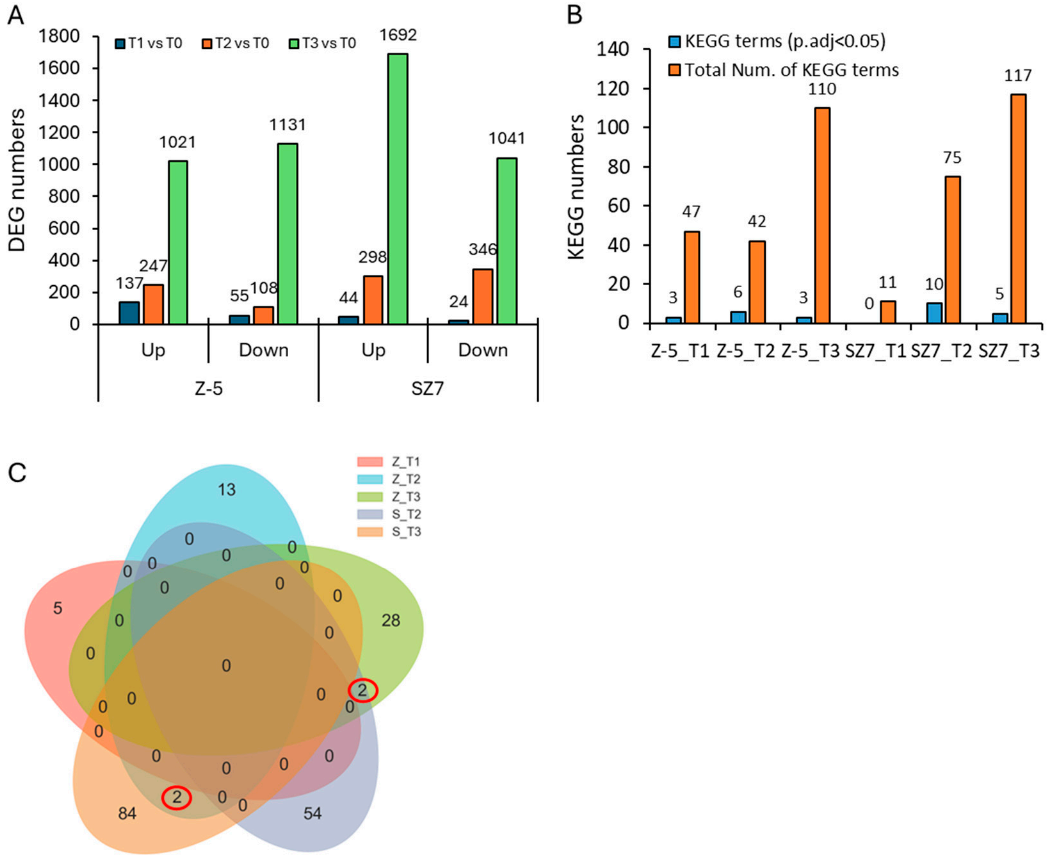

Figure S8 Integrated comparison of KEGG pathways for two varieties at different time intervals. A, DEG numbers totally enriched in KEGG pathways. B, Numbers of KEGG pathway enriched both in Z-5 and SZ7. C, Venn diagram of DEGs from KEGG enriched in two accessions at different time intervals.

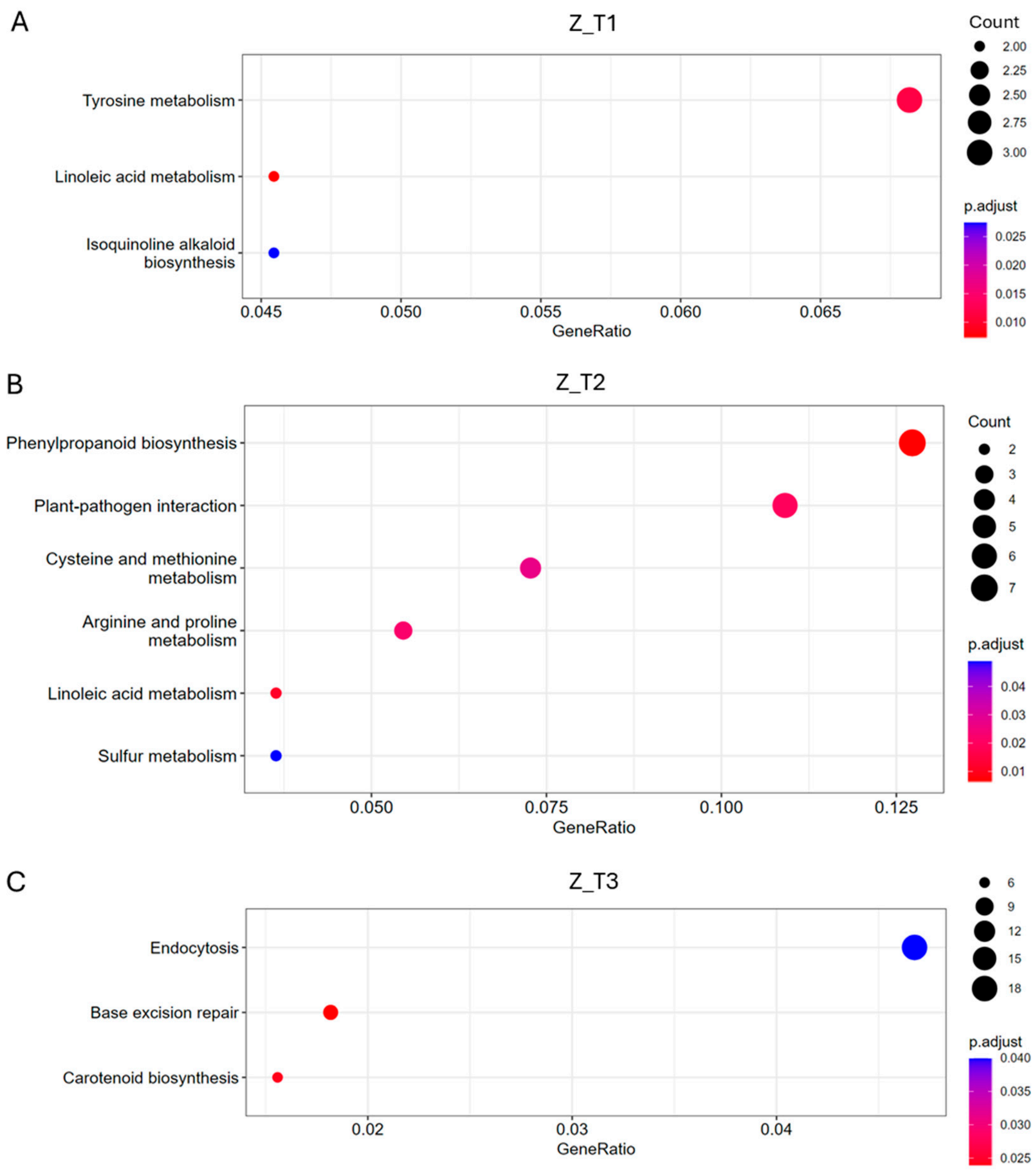

Figure S9 KEGG pathways significantly enriched in Z-5 at different time intervals. KEGG pathways significantly enriched in Z-5 at Z_T1 (A), Z_T2 (B) and Z_T3 (C), respectively. The X axes show enrichment scores. The terms with larger bubbles contain more DEGs. The bubble color changes from purple to red, indicating that the smaller the enrichment value, the greater the significance.

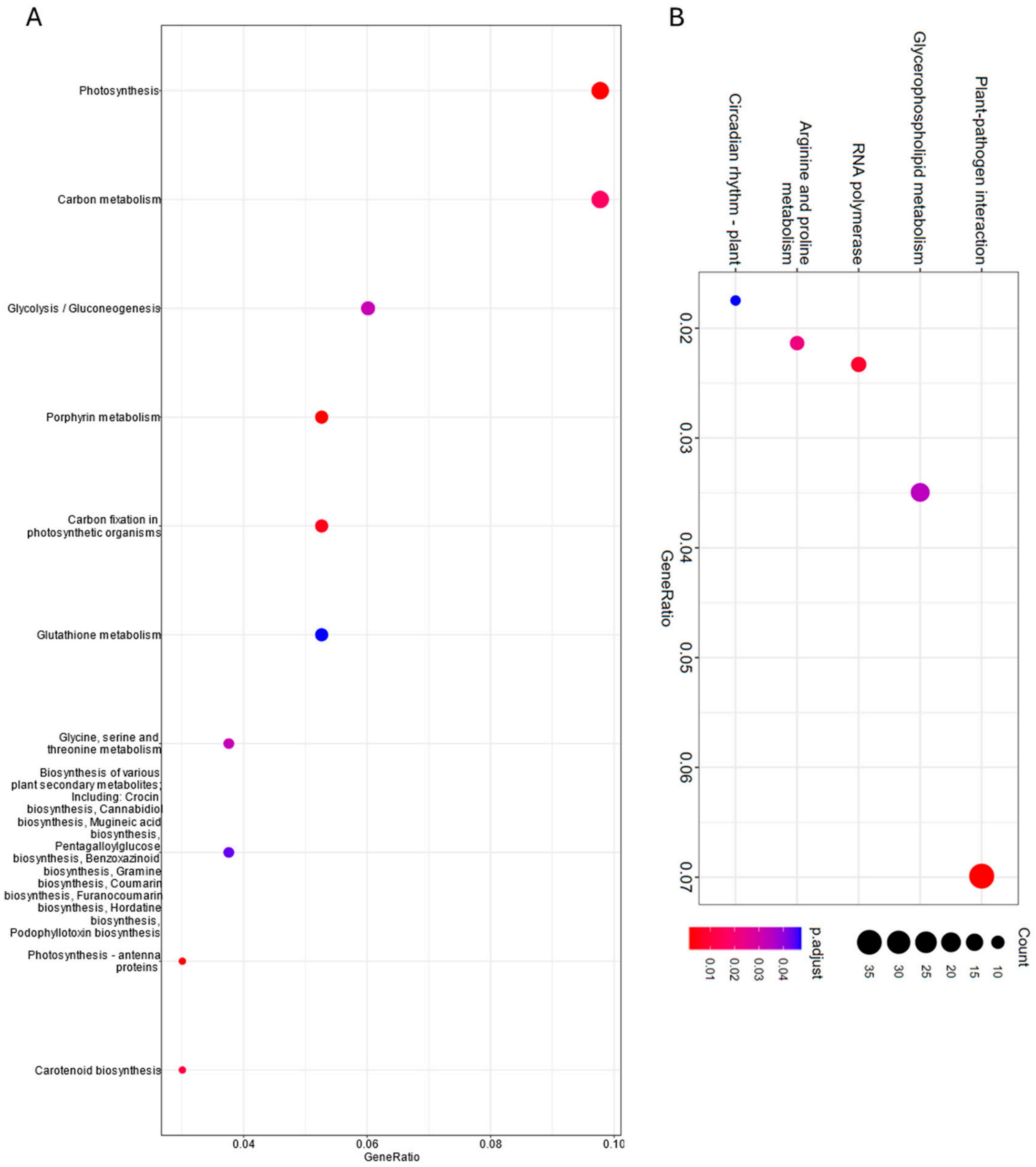

Figure S10 KEGG pathways significantly enriched in SZ7 at different time intervals. KEGG pathways significantly enriched in SZ7 at S_T2 (A), S_T3 (B), respectively. The X axes show enrichment scores. The terms with larger bubbles contain more DEGs. The bubble color changes from purple to red, indicating that the smaller the enrichment value, the greater the significance.

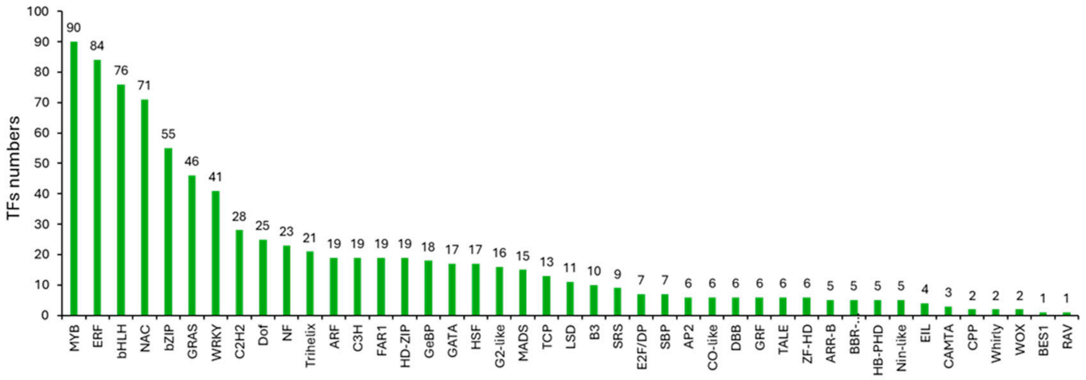

Figure S11 Statistics of TFs families and numbers significantly enriched both in Z-5 and SZ7.

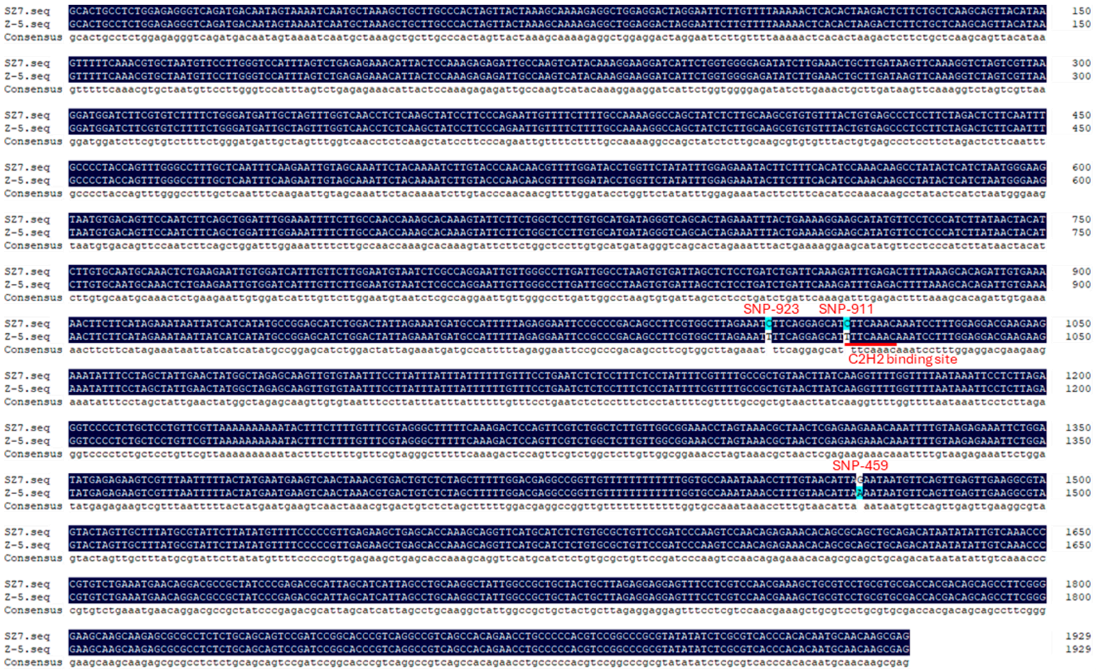

Figure S12 Alignment on promoter 2 k genome sequences of *SbERF027* between SZ7 and Z-5.
